# Prognostic Effects of Operation Age for Pediatric Patients with Supravalvar Aortic Stenosis

**DOI:** 10.31083/j.rcm2510384

**Published:** 2024-10-25

**Authors:** Lizhi Lv, Yuekun Sun, Simeng Zhang, Aihua Zhi, Cheng Wang, Qiang Wang

**Affiliations:** ^1^Department of Pediatric Cardiac Center, Beijing Anzhen Hospital, Capital Medical University, 100029 Beijing, China; ^2^Department of Cardiac Surgery, Yunnan Fuwai Cardiovascular Hospital, 650102 Kunming, Yunnan, China; ^3^Department of Cardiac Surgery, Peking University People’s Hospital, 100044 Beijing, China; ^4^Department of Radiology, Fuwai Hospital, National Center for Cardiovascular Diseases, Chinese Academy of Medical Sciences and Peking Union Medical College, 100037 Beijing, China; ^5^Department of Radiology, Yunnan Fuwai Cardiovascular Hospital, 650102 Kunming, Yunnan, China; ^6^Center for Pediatric Cardiac Surgery, Fuwai Hospital, National Center for Cardiovascular Diseases, Chinese Academy of Medical Sciences and Peking Union Medical College, 100037 Beijing, China

**Keywords:** supravalvular aortic stenosis, surgical intervention, operation age, inverse probability of treatment

## Abstract

**Background:**

The appropriate age for surgical repair of asymptomatic congenital supravalvular aortic stenosis (SVAS) is still unknown. The purpose of this research was to assess the safety and effectiveness of various operation ages when managing SVAS.

**Methods:**

Consecutive asymptomatic SVAS pediatric patients in the Beijing Fuwai and Yunnan Fuwai hospitals over a period of 18 years were retrospectively analyzed. Patients were classified as follows: age <2.0 years (y) (n = 84), 2.0–5.0 y (n = 72), and >5.0 y (n = 92). The primary safety endpoint was in-hospital death or extracorporeal membrane oxygenation (ECMO) needed. The primary effectiveness outcome was re-operation or restenosis during follow-up. To calculate the hazard ratios (HR), Cox regression with inverse probability of treatment weighted was utilized.

**Results:**

At the time of surgery, the median age of the 248 patients that were included was 4 y (interquartile range (IQR): 1.8–6.5). For the primary safety outcome, 7 (8.3%) patients in the age <2.0 y group had in-hospital death or ECMO needed, while no patients in the age 2.0–5.0 y and age>5.0 y groups (*p* = 0.001). The median follow-up was 25.5 months (IQR: 7.0–59.0). Compared with the age 2.0–5.0 y group, the age <2.0 y group and age >5.0 y group had a higher risk of re-operation or restenosis (age <2.0 y, HR = 3.27, 95% CI 1.25–8.60; age >5.0 y, HR = 2.87, 95% CI 1.19–6.91).

**Conclusions:**

Asymptomatic children with SVAS without other cardiovascular abnormalities should be considered for delayed surgical intervention until 2 years of age, and then surgery should be conducted as soon as possible. Children with severe symptoms should undergo surgery immediately, regardless of age.

**Clinical Trial Registration:**

ChiCTR2300067851, https://www.chictr.org.cn/showproj.html?proj=177491.

## 1. Introduction

Supravalvular aortic stenosis (SVAS) is a rare form of left ventricular outflow tract obstruction (LVOTO) that accounts for approximately 0.05% of all congenital heart diseases [1]. The natural history of SVAS is highly variable, determined by the type and degree of LVOTO, and is considered a progressive disease in children that may be associated with inadequate growth of the supravalvular aortic root and the sinotubular junction (STJ) [2, 3, 4]. For the majority of patients, SVAS is asymptomatic, but persistent LVOTO can lead to left ventricular hypertrophy if not treated surgically [5].

The age of surgery is currently not taken into account by most scholars [6]. The prevailing views were that the younger the age, the greater the risk of surgery and the greater the risk of reoperation due to the occurrence of restenosis [7, 8, 9, 10]. However, the delay of the operation could cause the persistence of left outflow tract obstructive lesions. High-velocity flow and high-pressure gradients of the bloodstream would lead to aortic valve degeneration and even left heart failure [11]. However, the appropriate age for surgical repair of asymptomatic SVAS is still unknown.

In this study, we reviewed patients with asymptomatic SVAS in two centers from March 2002 to April 2020. We analyzed the perioperative and postoperative mortality and complications, as well as the re-operation and restenosis rates during the follow-ups among different age groups to determine the optimal time to treat asymptomatic SVAS.

## 2. Materials and Methods

### 2.1 Patient Selection

**Fig. 1.**
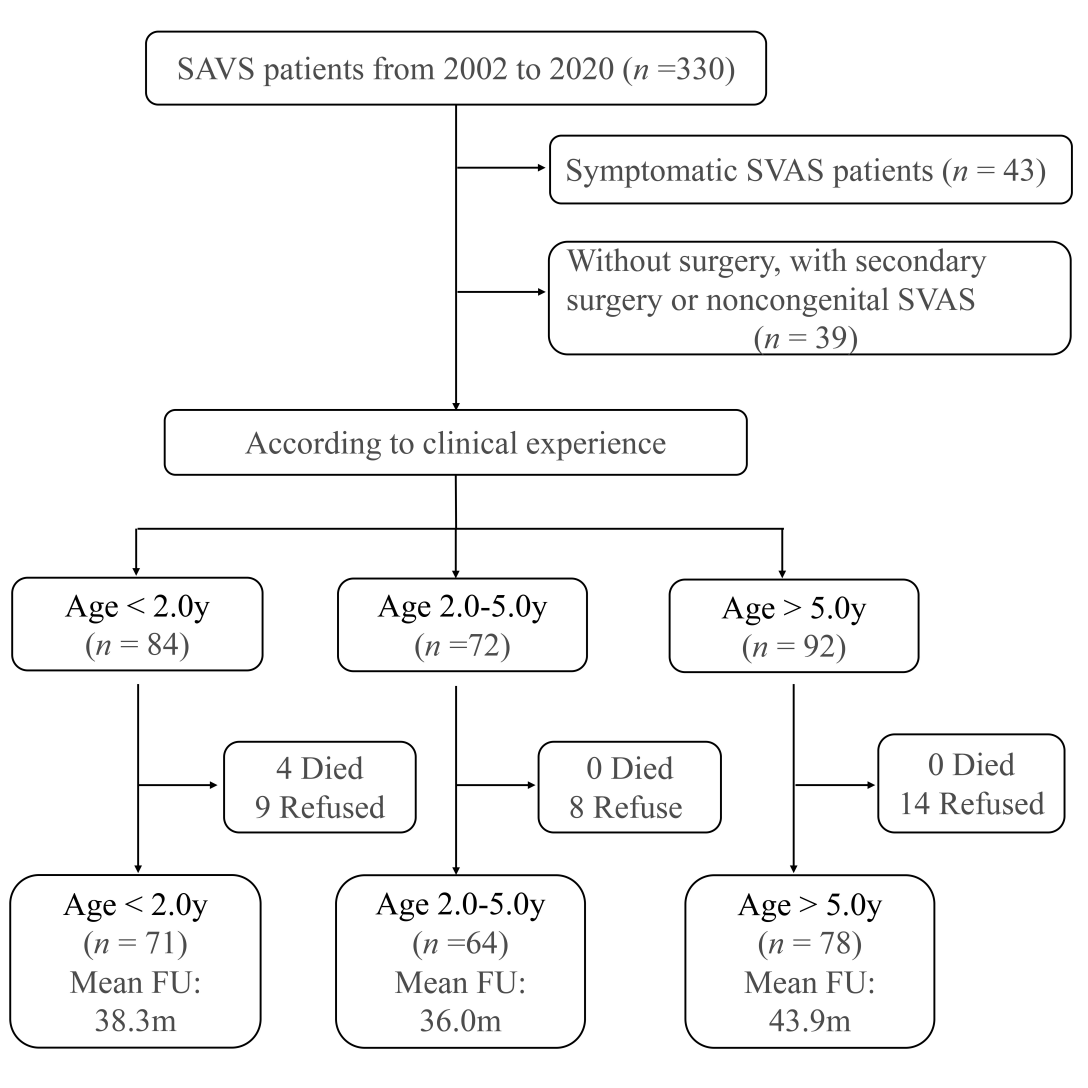
**Diagram illustrating the process of recruiting patients and follow-ups**. FU, follow-ups; SVAS, supravalvular aortic stenosis; y, years.

This retrospective cohort analysis comprised 330 consecutive patients who were admitted to Beijing Fuwai Hospital and Yunnan Fuwai Hospital between March 2002 and April 2020. Patients who were eligible had congenital SVAS, were under 18 years of age, and did not show any symptoms upon admission. SVAS was diagnosed by trans-thoracic echocardiogram. After excluding symptomatic SVAS patients, non-congenital SVAS patients, patients without surgery, and patients with secondary surgery, a final 248 patients were included. Patients were divided into three age groups according to clinical experience [12]. There were 84 patients aged <2.0 years, 72 patients aged 2.0–5.0 years, and 92 patients >5.0 years (Fig. 1). Our study was approved by the institutional ethics committee/institutional review board of Fuwai hospital (no.2021-1578). This study was registered at https://www.chictr.org.cn/showproj.html?proj=177491 (ChiCTR2300067851).

### 2.2 Variables and Outcomes

Cardiac surgery databases were used to collect baseline and follow-up information. The z-scores for the STJ and ascending aorta were computed using the echocardiography calculation tool provided by Boston Children’s Hospital.

The primary safety outcome was in-hospital death or extracorporeal membrane oxygenation (ECMO) needed. The primary effectiveness outcome was the occurrence of reoperation or restenosis which was characterized as average supravalvar aortic gradients that were more than 40 mmHg [1].

Secondary outcomes included re-operation, restenosis, surgery-related complications, supravalvar aortic gradients, STJ z-score, ascending aorta z-score, left ventricular ejection function (LVEF), and aortic valve regurgitation (AVR) during the follow-ups.

### 2.3 Statistics Analysis

Dichotomous variables were reported as the frequency with percentage. Continuous variables were reported as mean with standard deviation (SD) and median with interquartile range (IQR). In order to compare continuous variables that followed a normal distribution, analysis of variance was carried out, whereas the Kruskal-Wallis H test was applied to compare continuous variables that were not normally distributed. With the purpose of comparing categorical data, either the Pearson chi-squared test or Fisher’s exact test was conducted. For the primary effectiveness outcome, the freedom from restenosis and reoperation was shown with a Kaplan–Meier curve. The application of Cox regression models allowed for the computation of hazard ratios (HR). The inverse probability of treatment weighted (IPTW) method was utilized to evaluate the prognosis of various operation ages in order to avoid confounding bias as the primary analysis. The following conditions were used to determine propensity scores: gender, type, patent ductus arteriosus, bicuspid aortic valve, ascending aorta z-score, aortic valve regurgitation, pulmonary valve stenosis, pulmonary stenosis, and ventricular septal defect. The variable choice for propensity score was determined based on clinical expertise and ensuring baseline balance across groups. For the purpose of choosing the variable in the multivariate logistic regression models, the stepwise approach was used. Sensitivity analyses were performed to assess the reliability and stability of the primary analysis. Variables with a *p*-value < 0.1 in the univariate Cox regression model were selected in the multivariate model (**Supplementary Table 1**). Restricted cubic splines were used to test the non-linear association between operation age and the primary outcomes. Multiple imputation approaches were employed to fill in missing data. A two-sided *p*-value of less than 0.05 was deemed significant when making comparisons between the different groups. The Bonferroni correction was employed for multiple comparisons, with a significance level of 0.025 for a two-sided *p*-value considered statistically significant. The analyses were performed with R (version 4.0.3, R Foundation for Statistical Computing, Vienna, Austria).

## 3. Results

### 3.1 Baseline Information

The median age at operation was 4 y (IQR: 1.8–6.5) among 248 patients. Patients in the <2.0 y group had the greatest number of diffuse types (44 (52.4%)), Williams-Beuren syndrome (WBS) (36 (42.9%)), pulmonary stenosis (46 (54.8%)), pulmonary valve stenosis (33 (39.3%)), bicuspid aortic valve (7 (8.3%)), patent ductus arteriosus (12 (14.3%)), ventricular septal defect (14 (16.7%)), then followed by the 2.0–5.0 y group and finally the >5.0 y group. Patients in the 2.0–5.0 y group had the worst condition of ascending aorta z-score (–1.8 ± 1.5) compared with the other two groups. Patients in the >5.0 y group had more AVR (10 (10.9%)) compared with other groups. The proportion of female patients in the three groups was less than 40%. No difference was found in LVEF and STJ z-score among the three groups (Table 1). Natural history of SVAS could be evaluated in 28 of 248 patients suffering from this lesion (**Supplementary Fig. 1**).

**Table 1.** **Patients with asymptomatic SVAS: Baseline features and intraoperative data**.

Variables			Age <2.0 y (n = 84)	Age 2.0–5.0 y (n = 72)	Age >5.0 y (n = 92)	*p *value
Women			32 (38.1)	22 (30.6)	23 (25.0)	0.171
BSA (m^2^)			0.4 ± 0.1	0.7 ± 0.1	1.1 ± 0.4	<0.0001
			0.5 (0.4, 0.5)	0.7 (0.6, 0.7)	1.0 (0.8, 1.3)	
LVEF			69.2 ± 7.5	71.1 ± 6.9	69.8 ± 7.3	0.259
			69.7 (65.0, 72.5)	71.2 (65.0, 76.0)	69.7 (65.0, 75.0)	
Supravalvar aortic gradients (mmHg)			62.9 ± 29.6	74.9 ± 33.8	84.9 ± 34.6	<0.0001
			64.0 (42.5, 77.0)	71.0 (54.9, 88.4)	75.3 (66.0, 98.0)	
STJ z-score			–0.2 ± 1.5	–0.4 ± 1.1	–0.2 ± 1.1	0.497
			–0.4 (–1.2, 0.5)	–0.4 (–1.0, 0.2)	–0.2 (–1.0, 0.5)	
Ascending aorta z-score			–1.4 ± 2.3	–1.8 ± 1.5	–1.0 ± 2.2	0.026
			–1.8 (–3.1, –0.2)	–2.0 (–2.9, –1.1)	–1.3 (–2.4, –0.0)	
Type II (Diffuse)			44 (52.4)	32 (44.4)	19 (20.7)	<0.0001
WBS			36 (42.9)	20 (27.8)	6 (6.5)	<0.0001
PS			46 (54.8)	26 (36.1)	12 (13.0)	<0.0001
PVS			33 (39.3)	17 (23.6)	4 (4.3)	<0.0001
Ascending aortic stenosis			6 (7.1)	6 (8.3)	4 (4.3)	0.559
Bicuspid aortic valve			7 (8.3)	2 (2.8)	0 (0.0)	0.012
PDA			12 (14.3)	2 (2.8)	2 (2.2)	0.002
VSD			14 (16.7)	2 (2.8)	2 (2.2)	<0.0001
LVOTS			0 (0.0)	0 (0.0)	2 (2.2)	0.181
HOCM			0 (0.0)	1 (1.4)	2 (2.2)	0.414
MVS			1 (1.2)	1 (1.4)	2 (2.2)	0.861
MVR			8 (9.5)	6 (8.3)	7 (7.6)	0.900
AVS			14 (16.7)	15 (20.8)	17 (18.5)	0.800
AVR			1 (1.2)	4 (5.6)	10 (10.9)	0.026
Supra-aortic septum			1 (1.2)	2 (2.8)	3 (3.3)	0.653
Subaortic septum			5 (6.0)	1 (1.4)	5 (5.4)	0.325
Intra-operative						
	Surgical technique					0.919
		McGoon repair	42 (50.0)	34 (47.2)	45 (48.9)	
		Doty repair	34 (40.5)	33 (45.8)	41 (44.6)	
		Others	8 (9.5)	5 (6.9)	6 (6.5)	
	CPB (min)		126.6 ± 84.3	100.9 ± 47.4	100.5 ± 43.6	0.008
			103.0 (85.0, 138.0)	89.5 (72.5, 108.0)	87.5 (70.0, 116.0)	
	CCP (min)		78.6 ± 40.0	62.3 ± 27.6	67.2 ± 33.3	0.009
			72.0 (51.5, 92.5)	55.5 (45.0, 73.1)	56.0 (46.0, 80.0)	

AVR, aortic valve regurgitation; AVS, aortic valve stenosis; BSA, body surface area; CPB, cardiopulmonary bypass; CCP, cross-clamping; HOCM, hypertrophic obstructive cardiomyopathy; LVOTS, left ventricular outflow tract stenosis; MVR, mitral valve regurgitation; MVS, mitral valve stenosis; PDA, patent ductus arteriosus; PS, pulmonary stenosis; PVS, pulmonary valve stenosis; STJ, sinotubular junction; VSD, ventricular septal defect; WBS, Williams-Beuren syndrome; SVAS, supravalvular aortic stenosis; y, years; LVEF, left ventricular ejection fraction.

### 3.2 Operative and Postoperative Information

There was no statistically significant difference in the selection of surgical procedures across different groups (*p* = 0.919). Patients in the <2.0 y group had the longest cardiopulmonary bypass (CPB) time (126.6 ± 84.3 min) and cross-clamping (CCP) time (78.6 ± 40.0 min) compared with the 2.0–5.0 y (CPB 100.9 ± 47.4 min; CCP 62.3 ± 27.6 min) and >5.0 y group (CPB 100.5 ± 43.6 min; CCP 67.2 ± 33.3 min) (Table 1).

For the primary safety outcomes, patients aged <2.0 y (7 (8.3%)) had more in-hospital deaths or ECMO needed compared with patients aged 2.0–5.0 y or aged >5.0 y (*p* = 0.001). Six patients required postoperative ECMO assistance, three of whom died in the hospital. Cumulatively, four patients (1.4%) died, all treated with the McGoon repair, three of these deaths were attributed to heart failure while the remaining death was due to pulmonary arterial hypertension. For surgery-related complications, patients aged <2.0 y, had more repeated aortic clamping, delayed chest closure, and ECMO needed compared with the other two groups. Patients aged >5.0 y had more intraoperative defibrillation compared with the other two groups. During hospitalization, eleven patients had re-operation, nine of them were <2.0 years and the remaining were >5.0 years (Table 2).

**Table 2.** **Patients with asymptomatic SVAS: Post-operative data**.

Variables		Age <2.0 y (n = 84)	Age 2.0–5.0 y (n = 72)	Age >5.0 y (n = 92)	*p *value
Primary safety outcome					
	In-hospital death or ECMO	7 (8.3)	0 (0.0)	0 (0.0)	0.001
Surgery-related complications^1^		20 (23.8)	8 (11.1)	23 (25.0)	0.047
	Repeated aortic clamping	6 (7.1)	3 (4.2)	1 (1.9)	0.039
	Cardiac defibrillation	7 (8.3)	5 (6.9)	22 (23.9)	0.002
	Delayed chest closure	5 (6.0)	0 (0.0)	0 (0.0)	0.007
	ECMO	6 (7.1)	0 (0.0)	0 (0.0)	0.003
	Acute myocardial infarction	0 (0.0)	1 (1.4)	0 (0.0)	0.293
	Fatal arrhythmia	2 (2.4)	0 (0.0)	0 (0.0)	0.140
Post-operation					
	LVEF	66.3 ± 7.6	66.7 ± 5.3	67.9 ± 7.3	0.302
		65.0 (62.0, 71.0)	65.1 (64.0, 70.0)	68.0 (65.0, 72.5)	
	Supravalvar aortic gradients (mmHg)	17.3 ± 17.0	19.1 ± 15.3	21.9 ± 18.4	0.196
		11.0 (5.9, 22.5)	16.0 (6.7, 27.2)	18.2 (6.8, 29.2)	
	STJ z-score	1.8 ± 2.6	1.4 ± 1.7	1.4 ± 1.6	0.918
		1.1 (–0.2, 3.3)	1.3 (0.0, 2.4)	1.4 (0.3, 2.4)	
	Ascending aorta z-score	–0.4 ± 2.2	–0.7 ± 1.6	–0.8 ± 1.5	0.444
		–0.8 (–2.0, 0.4)	–0.6 (–1.8, 0.6)	–0.9 (–1.5, –0.0)	
	Postoperative pressure gradient ≥40 mmHg	6 (7.1)	8 (11.1)	14 (15.2)	0.239
	Re-operation during hospitalization	9 (10.7)	0 (0.0)	2 (2.2)	0.002
	In-hospital death	4 (4.8)	0 (0.0)	0 (0.0)	0.019

^1^Surgery-related complications includes repeated aortic clamping, cardiac defibrillation, delayed chest closure, ECMO assistance, acute myocardial infarction, fatal arrhythmia. ECMO, extracorporeal membrane oxygenation; LVEF, left ventricular ejection fraction; STJ, sinotubular junction; y, years.

### 3.3 Follow-up Information

85.9% (213/248) of patients had access to Echocardiographic follow-ups. There was no significant difference in the baseline information between patients who received follow-ups and those who did not. Follow-ups lasted for a median of 25.5 months (IQR: 7.0–59.0).

During the follow-ups, no death occurred and eight patients (3.8%) had re-operations. Aortic valvuloplasty was performed on two patients and Ross surgery was performed on another two patients. Two of the four remaining patients had simultaneous aortic arch reconstruction and valvuloplasty in addition to SVAS re-correction.

**Fig. 2.**
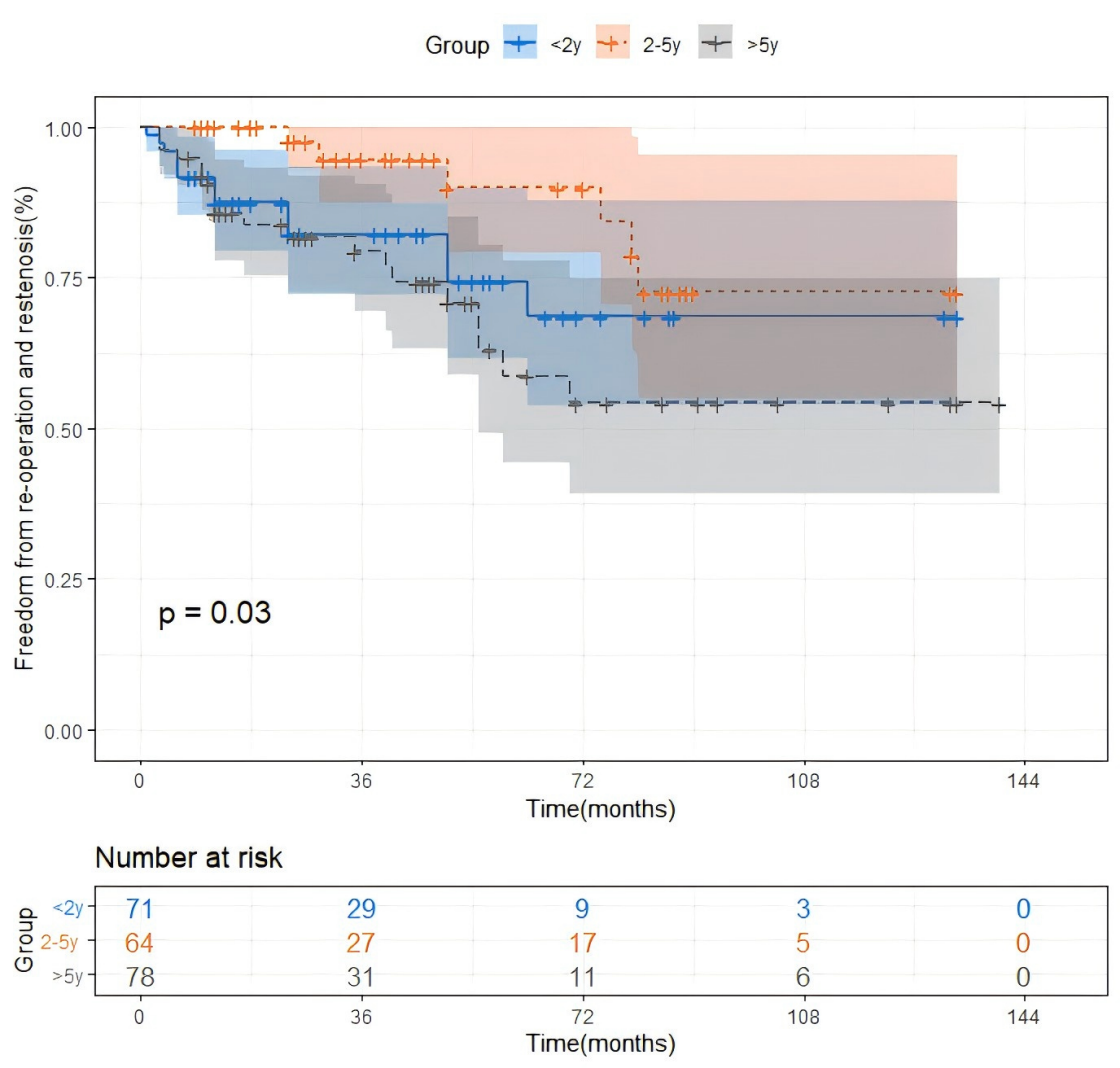
**Kaplan-Meyer estimates of re-operation or restenosis after surgery for supravalvular aortic stenosis (SVAS)**. The log-rank test revealed a significant difference across the groups (*p* < 0.05). y, years.

For the primary effectiveness outcome, patients aged <2.0 y (13 (18.3%)) or aged >5.0 y (20 (25.6%)) had more re-operation and restenosis compared with patients aged 2.0–5.0 y (6 (9.4%)) (Table 3). Kaplan–Meier survival curves were shown in Fig. 2.

**Table 3.** **Patients with asymptomatic SVAS: Follow-up data**.

Variables		Age <2.0 y (n = 71)	Age 2.0–5.0 y (n = 64)	Age >5.0 y (n = 78)	*p *value
Primary effectiveness outcome					
	Re-operation or restenosis	13 (18.3)	6 (9.4)	20 (25.6)	0.049
Secondary outcome					
	Re-operation	5 (7.0)	1 (1.6)	2 (2.6)	0.212
	Restenosis	8 (11.3)	6 (9.4)	18 (23.1)	0.031
	Supravalvar aortic gradients (mmHg)	17.6 ± 16.8	16.7 ± 18.5	28.3 ± 32.4	0.012
		10.2 (5.8, 21.2)	9.0 (4.8, 20.6)	19.4 (7.8, 36.0)	
	STJ z-score	1.6 ± 2.3	1.9 ± 2.3	1.9 ± 2.3	0.728
		1.2 (0.2, 2.9)	1.2 (0.1, 2.9)	1.8 (0.5, 3.2)	
	Ascending aorta z-score	–0.2 ± 2.3	–0.2 ± 2.1	–0.1 ± 2.3	0.922
		–0.3 (–1.6, 1.1)	–0.3 (–1.7, 1.1)	–0.5 (–1.5, 1.4)	
	LVEF	67.7 ± 4.7	66.7 ± 4.4	67.8 ± 5.3	0.359
		67.2 (65.0, 70.0)	65.5 (65.0, 70.0)	68.0 (64.0, 70.0)	
	Aortic valve regurgitation	3 (4.2)	4 (6.3)	7 (9.0)	0.553

LVEF, left ventricular ejection fraction; STJ, sinotubular junction; SVAS, supravalvular aortic stenosis; y, years.

### 3.4 Primary Analysis and Sensitivity Analyses

**Fig. 3.**
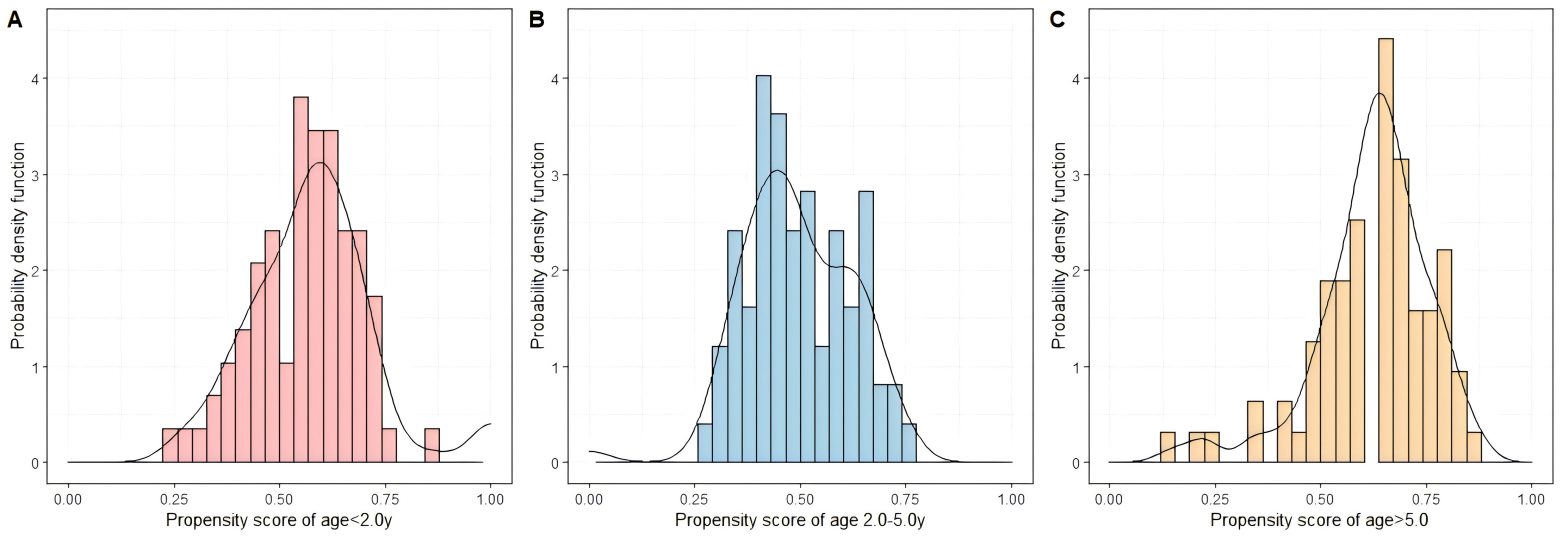
**Histogram and probability density function of the propensity score for three age groups**. (A) Age <2.0 y group. (B) Age 2.0–5.0 y group. (C) Age >5.0 y group. y, years.

The propensity score distributions for patients aged <2.0 y, 2.0–5.0 y, and >5.0 y were shown in Fig. 3. The primary analysis was conducted based on a propensity score (PS) used to calculate the weight for IPTW analysis. Compared with the age 2.0–5.0 y group, the age <2.0 y group and age >5.0 y group had more risk of re-operation or restenosis (age <2.0 y, HR = 3.27, 95% CI 1.25 to 8.60; age >5.0 y, HR = 2.87, 95% CI 1.19 to 6.91). The results were stable in sensitivity analyses, including multivariable plus PS adjusted logistic regression, and multivariable logistic regression, except that the age <2.0 y group had no significant difference of re-operation and restenosis risk in the crude model compared with the age 2.0–5.0 y group (Table 4).

**Table 4.** **Primary effectiveness outcomes of different age groups**.

		Age <2.0 y vs Age 2.0–5.0 y		Age >5.0 y vs Age 2.0–5.0 y	
		HR (95% CI)	*p* value	HR (95% CI)	*p *value
Primary analysis					
IPTW					
	Re-operation or restenosis	3.27 (1.25, 8.60)	0.016	2.87 (1.19, 6.91)	0.019
Sensitivity analysis					
Multivariable^1^ plus PS adjustment					
	Re-operation or restenosis	3.26 (1.16, 9.18)	0.025	3.26 (1.19, 8.94)	0.021
Multivariable^1^					
	Re-operation or restenosis	3.27 (1.18, 9.07)	0.023	3.47 (1.28, 9.41)	0.015
Crude models					
	Re-operation or restenosis	2.51 (0.95, 6.65)	0.063	3.31 (1.33, 8.26)	0.010

^1^The models were adjusted for gender, STJ z-score, type of supravalvular aortic stenosis and Williams-Beuren syndrome, pulmonary stenosis and bicuspid aortic valve. CI, confidence interval; IPTW, inverse probability of treatment weighted; PS, propensity score; HR, hazard ratios; y, years.

**Fig. 4.**
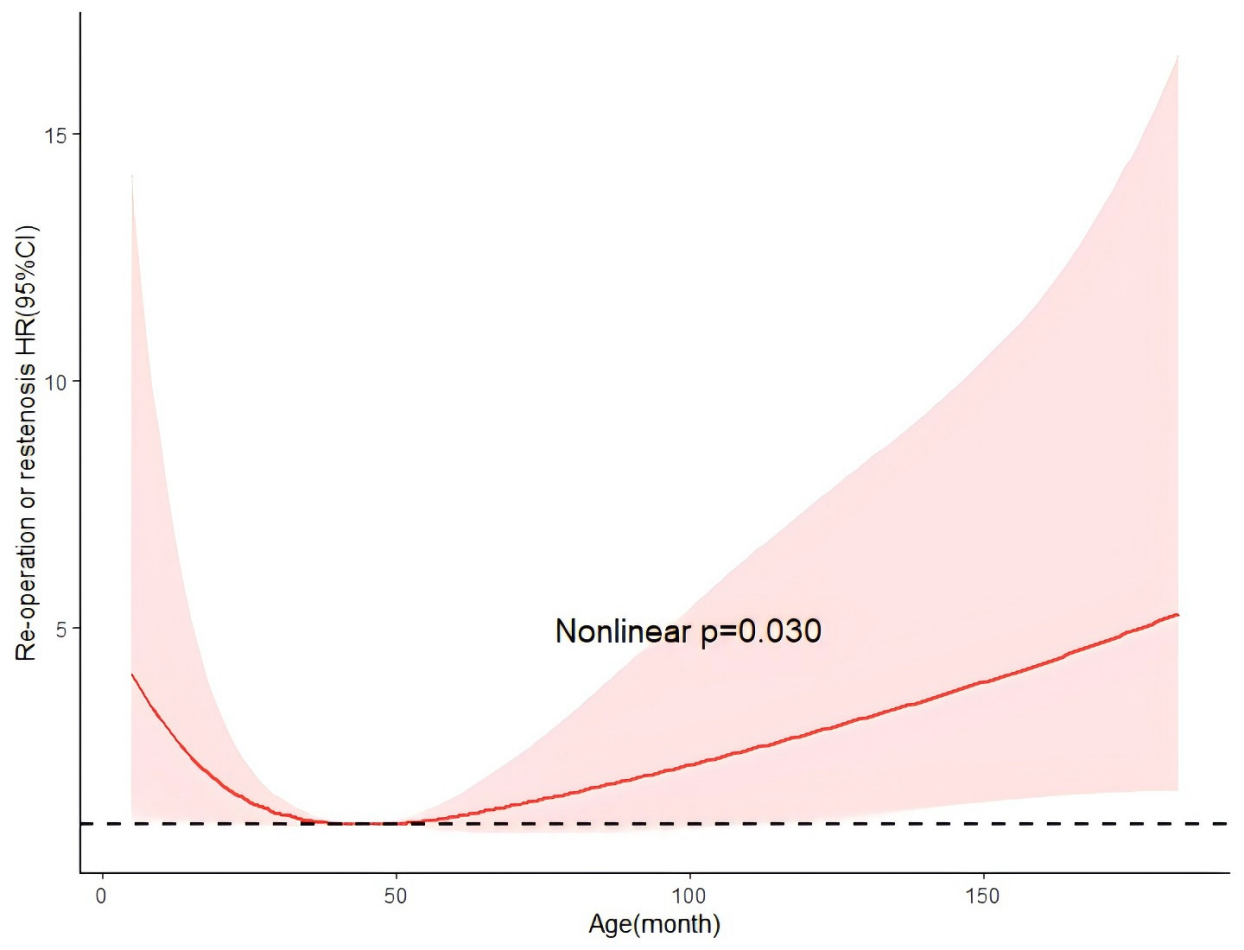
**Restricted spline curve for the association between operation age and re-operation or restenosis**. The models were adjusted for gender, STJ z-score, type, and Williams-Beuren syndrome, pulmonary stenosis, and bicuspid aortic valve; STJ, sinotubular junction; HR, hazard ratios; CI, confidence interval.

The restricted cubic splines showed the non-linear regression between age and the primary effectiveness outcomes and the results indicate that an age around 48 months (4 years) exhibited a decreased rate of reoperation or restenosis (Fig. 4).

## 4. Discussion

This study first explored the operation age in patients with asymptomatic SVAS. Patients aged <2.0 years had significantly higher in-hospital death or were more likely to need ECMO compared to the other two groups. Patients who underwent surgical treatment at <2.0 years or >5.0 years of age showed a higher risk of restenosis or re-operation compared with patients aged 2.0–5.0 years. The nonlinear analysis indicated that patients aged 4 years had the lowest restenosis or re-operation risk.

The most common symptoms of pediatric patients for SVAS were dyspnea on exertion, angina, or syncope. However, the vast majority were asymptomatic, and only 7% had New York Heart Association (NYHA) class IV symptoms [13]. The early report described only 1.2% of pediatric patients with SVAS having NYHA class IV symptoms [14]. The median age at surgery of the included patients in our cohort was 4 years (IQR: 1.8–6.5). This was consistent with other previous single-center large cohort studies, which reported a median age at surgery of 3.3 or 4.3 years [8, 9]. Our cohort had a similar age distribution with other studies and had good representation.

In our cohort, the McGoon and Doty repairs were mainly used for the treatment of SVAS. For both procedures, we did not find differences in the reduction of pressure gradients and the risk of re-operation in the medium term in our previous studies. And this finding was similar to previous studies [15, 16, 17]. We used the Brom repair in 3 children, but we had little experience with this technique and all that was demonstrated was a significantly higher CPB and CCP. Nonetheless, the overall surgical repair described in this study had a good outcome after surgery, and the early postoperative mortality was lower than in previous studies [15].

We divided the enrolled asymptomatic SVAS children into early childhood (<2.0 years), younger children (2.0–5.0 years), and older children (>5.0 years) based on clinical experience. We found that all early deaths and ECMO occurred in patients <2.0 years and the risk of re-operation or restenosis was significantly higher than in the other two groups during follow-ups. Surgical repair in early childhood was challenging, and patients receiving surgical intervention younger than 2 years were often at higher risk of re-intervention [7, 8, 9, 10, 13, 18]. Previous studies also demonstrated that the majority of surgical deaths occurred in children who were younger than 2 years [6, 8]. The smaller aortic root size and smaller coronary lumen size in younger patients might make SVAS repair more difficult but there was no evidence directly suggesting that these factors affect early and late outcomes. Associated concomitant conditions described in these deaths included diffuse disease, severe biventricular obstruction, coronary artery involvement, and combinations of these lesions. Coronary artery stenosis, coronary ostial ectopia, and progressive damage to the aortic root are all compelling justifications for surgical intervention in patients with severe SVAS. Although children with severe anatomical lesions inevitably required early and urgent intervention, our study warned that surgeons need to be more vigilant in these patients because they had a higher surgery risk.

For older children, the surgery is reliably safe, but persistent LVOTO leads to left ventricular hypertrophy and impairment of aortic valve function. Previous studies have indicated that the natural history of SVAS in adolescent patients is progressive, with age correlating with an increase in transvalvular pressure gradients [2, 3, 4, 5]. Our study has also demonstrated similar findings. The most important late complication of SVAS is AVR, and it is usually progressive [19]. In our study, the rate of preoperative combined AVR was 10.9% in the older children group, which was significantly higher than in the other two groups. A natural history study [20] found that the presence of AVR indicated the need for surgical intervention. Moreover, AVR is a major sequela in patients with SVAS and has significant prognostic implications. A study showed that a higher left ventricular mass index in adult patients undergoing SVAS surgery was associated with more adverse cardiovascular events and a greater need for re-operation [21]. In this study, the risk of restenosis during follow-up was significantly higher in the older children than in the other two groups, and it is inconclusive whether this is due to the presence of left ventricular hypertrophy leading to a high transvalvular pressure gradient after SVAS repair. Some authors have argued that early prophylactic intervention for LVOTO is not beneficial and therefore unnecessary [22]. However, due to the progressive nature of the disease [2], some authors recommend not delaying intervention for SVAS and not waiting until irreversible damage is caused [23]. In addition, there was evidence that surgical repair to reduce aortic gradients might help improve left ventricle remodeling [24]. Therefore, intervention for SVAS should not be recklessly delayed and trigger other abnormalities of cardiac function, delaying the best time for treatment.

This study compared the prognosis of the different age groups based on a large sample study with a low drop rate, and for the first time suggested the optimal age of surgery for SVAS repair. Nevertheless, there were still some limitations. Firstly, this was neither a randomized controlled trial nor prospective cohort research. However, we made adequate adjustments for possible confounding factors and conducted sensitivity analyses to test the robustness of the results. Secondly, we did not include patients without an operation, and the prognosis between patients with or without SVAS operation in the same age group was not analyzed.

## 5. Conclusions

With advances in medicine, ensuring the quality of life for children with congenital heart disease has become a tendency. Definitive surgical repair is the best option for patients with SVAS, but early treatment has high surgical safety issues and risk of late re-operation, and too late intervention may compromise aortic valve function while causing irreversible damage to cardiac function. The optimal age for surgical repair of SVAS was an underappreciated issue in pediatric cardiac surgery. Based on this we found satisfactory overall results for the surgical repair of SVAS. Asymptomatic children with SVAS without associated risk of sudden cardiac death could be considered for delayed surgical intervention until 2 years of age, and then surgery should be conducted as soon as possible. Children with severe symptoms should undergo surgery immediately, regardless of age.

## Data Availability

The datasets generated during and/or analyzed during the current study are not publicly available due this dataset will continue to be used in subsequent studies but are available from the corresponding author upon reasonable request.

## Abbreviations

AVR, aortic valve regurgitation; ECMO, extracorporeal membrane oxygenation; LVOTO, left ventricular outflow tract obstruction; NYHA, New York Heart Association; STJ, sinotubular junction; SVAS, supravalvular aortic stenosis; WBS, Williams-Beuren syndrome.

## Supplementary Material

Supplementary material associated with this article can be found, in the online version, at https://doi.org/10.31083/j.rcm2510384.
