# Supplementary material for: Prognostic Effects of Operation Age for Pediatric Patients with Supravalvar Aortic Stenosis
Source: Rev Cardiovasc Med. 2024 Oct 25;25(10):384. doi: 10.31083/j.rcm2510384 (PMC11522753; doi:10.31083/j.rcm2510384)
Supplement: Supplementary file 1 [file 2153-8174-25-10-384-s1.zip › Supplementary material.docx]

Prognostic Effects of Operation Age for Pediatric Patients with Supravalvar Aortic Stenosis

Supplementary Table 1. Results of univariate and multivariate cox regression model.

|  | **Univariate** | | **Multivariate** | |
| --- | --- | --- | --- | --- |
|  | **HR (95%CI)** | ***p* Value** | **HR (95%CI)** | ***p* Value** |
| Men | 2.38 (0.98, 5.80) | 0.056 | 1.15 (0.46, 2.83) | 0.766 |
| **Echocardiographic information** | | | | |
| STJ gradients (mmHg) | 1.01 (0.89, 1.02) | 0.393 |  |  |
| STJ z-score | 0.64 (0.51, 0.82) | <0.001 | 0.62 (0.48, 0.81) | <0.001 |
| Ascending aorta z-score | 1.08 (0.89, 1.32) | 0.444 |  |  |
| Type II (Diffuse) | 2.63 (1.14, 5.88) | 0.024 | 1.22 (0.37, 4.01) | 0.745 |
| WS (Yes) | 3.45 (1.16, 10.00) | 0.026 | 1.15 (0.46, 2.83) | 0.766 |
| **Concomitant cardiovascular anomalies** | |  |  |  |
| PS (Yes) | 4.00 (1.19, 14.29) | 0.025 | 3.13 (0.23, 11.30) | 0.385 |
| PVS (Yes) | 2.63 (0.08, 5.26) | 0.668 |  |  |
| Bicuspid aortic valve (Yes) | 2.55 (1.00, 6.47) | 0.049 | 2.32 (0.84, 6.40) | 0.105 |
| PDA (Yes) | 1.80 (0.17, 3.77) | 0.779 |  |  |
| VSD (Yes) | 1.30 (0.04, 2.36) | 0.253 |  |  |
| AVR (Yes) | 1.23 (0.33, 4.65) | 0.755 |  |  |

AVR = Aortic valve regurgitation; AVS = Aortic valve stenosis; PDA = Patent ductus arteriosus; PS=Pulmonary stenosis; PVS=Pulmonary valve stenosis; STJ=Sinotubular junction; VSD = Ventricular septal defect; WS=Williams syndrome.


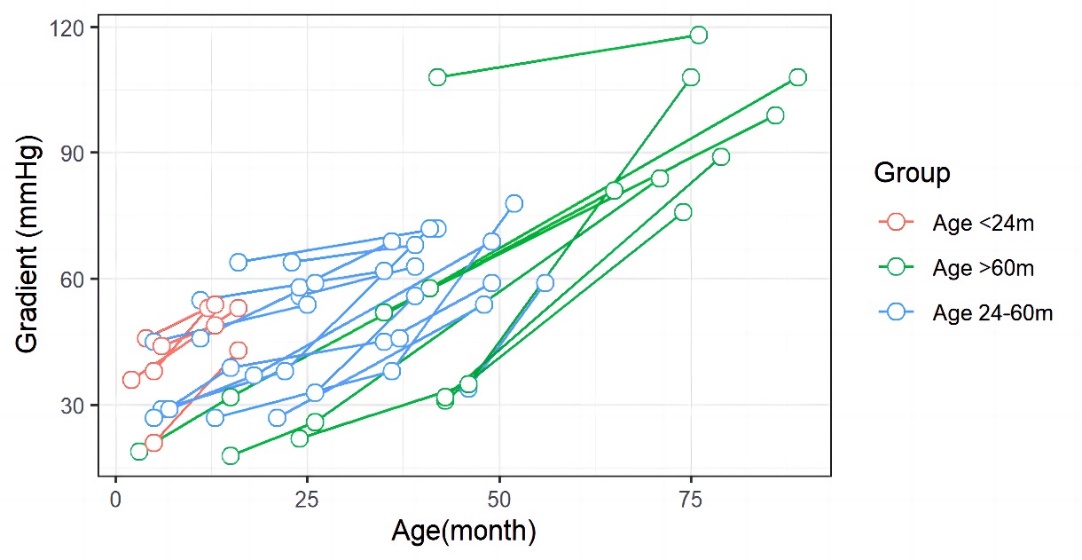


**Supplementary Fig. 1. Natural history of supravalvar aortic gradients at supravalvular aortic stenosis (SVAS).** First and last individual measurement connected by line. Red circles indicate patients undergoing surgery <24 months of age. The blue circles indicate patients patients undergoing surgery between 24 and 60 months of age. The green circle represents patients undergoing surgery >60 months of age.
