# Supplementary figures and images for: Prognostic Effects of Operation Age for Pediatric Patients with Supravalvar Aortic Stenosis
Source: Rev Cardiovasc Med. 2024 Oct 25;25(10):384. doi: 10.31083/j.rcm2510384 (PMC11522753; doi:10.31083/j.rcm2510384)

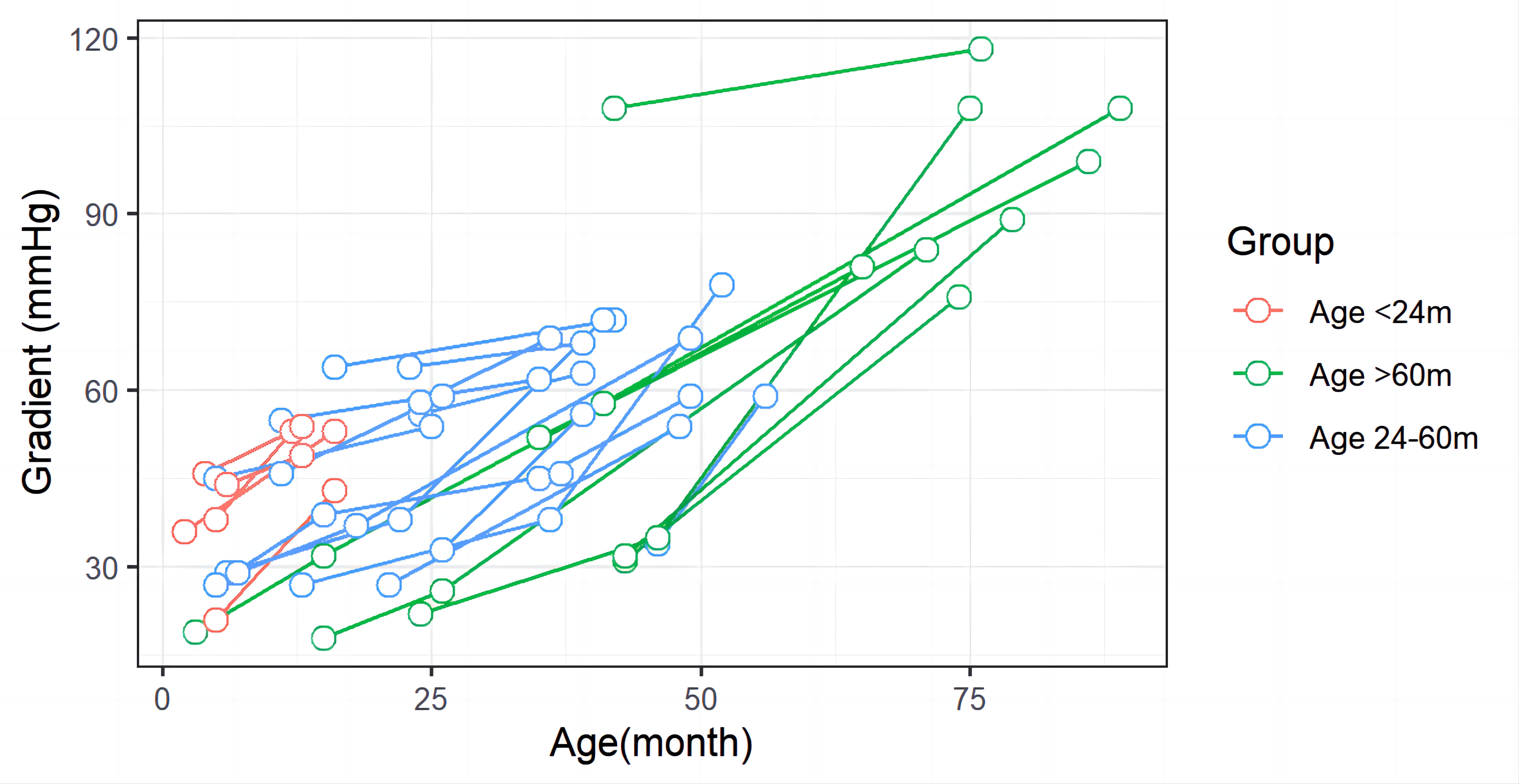

Supplement: Supplementary file 1 [file 2153-8174-25-10-384-s1.zip › Supplementary Fig. 1.jpg]
